# Landscape assessment of the availability of medical abortion medicines in India

**DOI:** 10.1186/s12978-024-01774-5

**Published:** 2024-06-05

**Authors:** Priya Karna, K. Aparna Sharma, Amy Grossman, Madhur Gupta, Tapas Chatterjee, Natalie Williams, Ndola Prata, Annik Sorhaindo, Laurence Läser, Ulrika Rehnström Loi, Bela Ganatra, Pushpa Chaudhary

**Affiliations:** 1 World Health Organization, Country Office for India, New Delhi, India; 2 Department of Obstetrics and Gynecology, All India Institute of Medical Science (AIIMS), New Delhi, India; 3 Venture Strategies for Health & Development/OASIS, Berkeley, CA USA; 4 Bixby Center for Population, Health & Sustainability, School of Public Health, University of California, Berkley, CA USA; 5 UNDP‑UNFPA‑UNICEF‑WHO‑World Bank Special Programme of Research, Development and Research Training in Human Reproduction (HRP), Department of Sexual and Reproductive Health and Research, World Health Organization, 20 Avenue Appia, 1211 Geneva, Switzerland

**Keywords:** Assessment, Availability, Quality, Abortion, Misoprostol, Mifepristone, Combi-pack, India

## Abstract

**Background:**

Medical abortion with mifepristone and misoprostol can be provided up to 63 days’ gestation in India. This accounts for 67.5 percent of all abortions in the country. We conducted an assessment to determine the availability of medical abortion medicines, specifically the combi-pack, in India.

**Methods:**

We applied the World Health Organization landscape assessment protocol at the national level. The assessment protocol included a five-step adaptation of an existing availability framework, including online data collection, desk review, country-level key informant interviews, and an analysis to identify barriers and opportunities to improve medical abortion availability. The assessment was conducted between August and March 2021.

**Results:**

Medicines for medical abortion are included in the national essential drug list and available with prescription in India. The assessment identified 42 combi-pack products developed by 35 manufacturers. The quality of medical abortion medicines is regulated by national authorities; but as health is devolved to states, there are significant inter-state variations. This is seen across financing, procurement, manufacturing, and monitoring mechanisms for quality assurance of medical abortion medicines prior to distribution. There is a need to strengthen supply chain systems, ensure consistent availability of trained providers and build community awareness on use of medical abortion medicines for early abortions, at the time of the assessment.

**Conclusion:**

Opportunities to improve availability and quality of medical abortion medicines exist. For example, uniform implementation of regulatory standards, greater emphasis on quality-assurance during manufacturing, and standardizing of procurement and supply chain systems across states. Regular in-service training of providers on medical abortion is required. Finally, innovations in evidence dissemination and community engagement about the recently amended abortion law are needed.

## Plain language summary

Medical abortion is popular in India and benefits from a liberal legal context. It is important to understand the availability of quality abortion medicines in the country. Using the World Health Organization country assessment protocol and availability framework for medical abortion medicines we examined the availability of these medicines from supply to demand. We used this information to identify opportunities for increasing availability of quality-assured medical abortion medicines. We found that the context for medical abortion varies across states. Strengthening procurement and supply chain management, with a greater emphasis on quality-assurance and regulation of manufacturing should be instituted at the state-level. Training is also needed to increase provider knowledge of the latest national guidelines and laws to ensure respectful and person-centered services. Finally, the public should be informed about medical abortion as a safe and effective choice, especially for early abortions.

## Background

Abortion has been legal in India for nearly five decades, accommodating a broad range of conditions [1]. Medical abortion (MA) using either a combination of mifepristone followed by misoprostol, or misoprostol alone is a well-accepted, safe, and effective method [2, 3]. As per the 2019–2021 National Family Health Survey (NFHS), MA is the predominant method of abortion in India (67.5%) [4]. Women’s preference for MA is influenced by various factors, like safety and effectiveness, degree of medical intervention, perception of what is natural, perceived pain and adverse effects, time required at the facility, confidentiality, need for multiple clinic visits, associated cost and physical examination requirements [5, 6]. Nearly half of women (48%) sought abortion due to unplanned pregnancy [6]. The World Health Organization (WHO) estimates that nearly 21.5 million or 44 percent of all pregnancies in India are unintended [7]. The share of unintended pregnancies that end in abortion has nearly doubled from 47 percent in 1990–1994 to 77 percent in 2015–2019 [7, 8].

WHO defines unsafe abortion as terminating an unintended pregnancy by unskilled individuals or in substandard medical conditions, or both [9]. In India, around 55% of abortions are performed by medical doctors, with a significant variation between rural (48%) and urban (66%) areas [4]. This indicates that rural areas are more susceptible to unsafe abortion practices compared to urban areas [6]. Women’s age, geographic location, gender composition of their living children, and their level of education are crucial predisposing factors influencing unsafe abortion in India [6]. A significant 27% of abortions in India are conducted at home. Notably, self-administered abortions account for 21.6% in urban areas, which starkly contrasts with the 30% in rural settings [4]. Therefore, the importance and potential scope of the use of MA medicines cannot be understated.

In India, health products are governed by the Drugs & Cosmetics Act [10], which covers a wide variety of medicines and medical devices. Mifepristone was approved under this act in 2002. In 2008, the Government of India (GoI) approved the use of co-packaged mifepristone and misoprostol products (combi-pack) for use up to nine weeks (63 days) of pregnancy. As a result of the introduction of MA medicines and subsequent widespread availability, the abortion landscape in India has changed substantially. Both mifepristone and misoprostol are considered ‘Schedule H drugs’ according to the Drugs and Cosmetics Act [10]. This means that these drugs require a written prescription by a registered medical practitioner (RMP). The specific characteristics of RMP are described within the Medical Termination of Pregnancy (MTP) Act Rules [1]. The definition of RMPs was broadened in the 2021 amendment that extended the scope of comprehensive abortion care (CAC) service provision [1, 11]. The National Comprehensive Abortion Care Training and Service Delivery Guidelines of 2018 and 2023 provide clinical guidance for MA use [12].

In recent years, the government has invested significantly in improving the quality of medicines and strengthening its regulatory agencies at the national and state-level [13]. In stark contrast to MA drugs, contraceptives in India are supplied via a centralized system, the FP-LMIS (Family Planning Logistics, Management, and Information System). MA medicines manufacturing, procurement and distributions are decentralized and determined at the state-level. Despite the high use of MA medicines and potential for growth [4], we found no previous comprehensive assessment of this landscape. Given India’s increasing focus on strengthening its overall pharmaceutical manufacturing capacity, through this assessment, we wanted to map all available MA medicines, and identify strategic areas of intervention to highlight the unique opportunity to position the country as a leading provider of MA medicines. Given this context, it is critical to understand the landscape of MA medicines, the mechanisms for quality assurance, barriers for use and opportunities to improve access to MA medicines in India.

The objectives of the assessment were to systematically identify the regulatory landscape including manufacturing, quality assurance standards, policy and financial norms governing availability of MA medicines in the market, both nationally and at the state-level. We aimed to better understand the procurement, storage, distribution, and overall use of MA medicines whilst also reviewing the service providers knowledge and end users' awareness regarding MA. This paper also identifies opportunities for increasing availability of quality-assured MA medicines. For our paper, a quality-assured medicine was defined as one that is either WHO Prequalification (WHO PQ)-listed or approved by a Stringent Regulatory Authority (SRA-approved) [14–16].

This assessment was completed before the MTP amendment of 2021, but opportunities identified through this assessment are relevant for the implementation of the CAC program especially after the law change [11].

## Methods

**Fig. 1:**
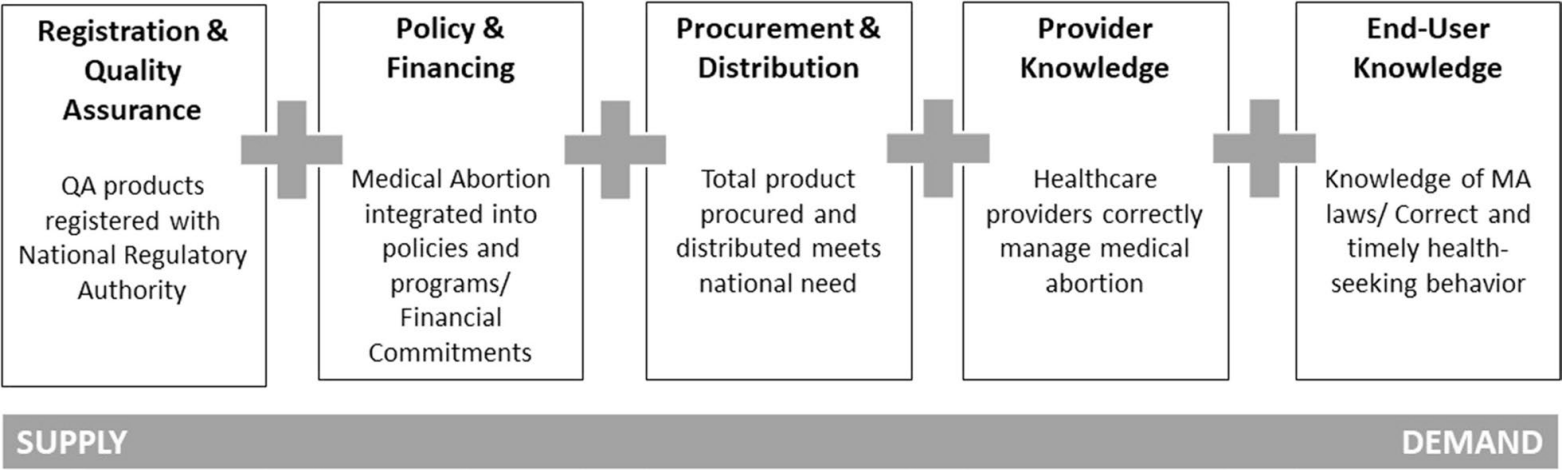
The five pillars of availability of a service related to a medical. Source: Rehnström Loi, U., Prata, N., Grossman, A. et al. In-country availability of medical abortion medicines: a description of the framework and methodology of the WHO landscape assessments. Reprod Health 20 (Suppl 1), 20 (2023). https://doi.org/10.1186/s12978-022-01530-7

We applied the WHO country assessment protocol for MA medicines at the country-level. The assessment protocol included five steps: (1) adaptation of availability framework as per country context, (2) literature review (3) country-level key informant interviews, (4) analysis of publicly available data to identify barriers and opportunities in MA medicines availability, and (5) validation of findings by the technical advisory group (Fig. 1). Each step has a set of conditions that should be fulfilled to ascertain MA medicine availability and span across all aspects of use, from supply by the manufacturer to demand and use by the end user [17].

As part of this assessment, we also conducted a deep dive in two states, Rajasthan, and Tamil Nadu, to better understand the state-level differences. The state-level analysis has not been included in this paper; however, the full report is available on the WHO website. The rationale for the state selection was significant utilization of MA drugs and presence of a well-established medical service corporation.

We conducted a comprehensive desk review to collate and analyse data from secondary sources, including government reports, National Family Health Surveys, state-level program implementation plans, published research articles and evaluation reports. Primary data was collected through 45 key informant interviews representing diverse stakeholder perspective, ranging from officials from the Ministry of Health and Family Welfare (MoHFW); the Federation of Obstetric and Gynaecological Societies of India; manufacturers and distributors and including social marketing organizations; academics; clinicians; and non-governmental organizations, as well as State Health Mission representatives from two states, Tamil Nadu, and Rajasthan.

The assessment was conducted between August and November 2020 and the findings were finalized in March 2021 following validation by national experts. The assessment occurred within the context of an ongoing national dialogue on abortion laws and policies. During this period, the MTP Act of 1971 was amended and ratified as “The MTP (Amendment) Act 2021”, unrelated to the assessment itself [11].

## Results

### Registration and quality assurance

#### Regulation

The Central Drugs Standard Control Organization (CDSCO), operating under the Director General of Health Services, MoHFW, serves as the primary national regulatory authority (NRA) in India. India is an active member of the Southeast Asia Regulatory Network, which seeks to increase access to high-quality medicines including MA products in the WHO member states in the region [18, 19].

In India, new medicines are initially registered, reviewed and then centrally approved by the drug controller under CDSCO with a restricted license issued for the period of three years [20, 21]. If the product meets quality and compliance standards consistently over three years, the restricted license transitions to a general license, and manufacturers can seek registration of their products with the state-level Food and Drug Control Administrations (FDCA). These FDCAs are then responsible for regulation and manufacturing of MA medicines in their state. Once a product is approved by either CDSCO or state-level FDCA, it is eligible for marketing and distribution throughout the country. This also applies to MA medicines [10].

#### Manufacturing

CDSCO manages an e-portal named SUGAM [21], which offers the up-to-date data on approvals, licenses, and details of all medicines manufacturing facilities including information on formulations and prescribed use for Schedule H drugs. The current licensed drug list of CDSCO includes misoprostol (Sr. No. 869) which is approved for “cervical ripening, prevention of postpartum hemorrhage and first trimester abortion along with mifepristone”. The list also includes mifepristone (Sr. No. 1039) approved for use “by Gynecologist only—for medical termination of intrauterine pregnancy through 49 days of inception” and a combi-pack of mifepristone + misoprostol (Sr. No. 1782) “for the medical termination of intrauterine pregnancy of up to 63 days gestation based on the first day of the last menstrual period” [21].

We identified 42 combi-pack products by 35 manufacturers available in India discerned through manufacturers’ and social marketers’ websites and online pharmacies (Table 1). These products are approved by a state-level FDCAs based upon the initial combi-pack approved by CDSCO in 2008 (Sr. No. 1782), however, their exact registration status within the country could not be verified. These 35 combi-packs manufacturers are distributed nationwide, with some operating in multiple states. Two misoprostol products manufactured in India are WHO PQ-listed and one combi-pack is SRA-approved; all three products are intended for export and manufactured in dedicated facilities (Table 1). There is an opportunity for the local manufacturers of MA medicines towards achieving WHO-PQ, which can elevate their global contribution, enhancing access to quality MA medicines.

**Table 1:** Medical Abortions (MA) Medicines Available in India

MA medicines in India	
*Manufacturer*	*Commercial name*
Combi-pack	
Acekinetics Healthcare Pvt Ltd	Abokit
Acme Formulations Ltd. export only	Mariprist/Mifiso
Alkem Laboratories Ltd	Termipil Kit
Aristo Pharmaceuticals Pvt Ltd	MiftyKit
Astronova Biotech	MizopristKit
Cadila Pharmaceuticals	Contrakit/Contrapill
Cipla Ltd	MTPKit/MTProstKit
Galpha Laboratories Ltd	Rimover Kit
Intas Pharmaceuticals	AntiPregKit
Johnlee Pharmaceuticals Pvt Ltd	Mitotec
Leeford Healthcare Ltd	Festone Combi Kit
Lifekyor Pharmaceuticals	Kyor
Lupin Ltd	PregNot Kit
Macleods Pharmaceuticals	InstaKit/Gestarest
Mankind Pharma Ltd	Unwanted Kit/Undezire Kit
Maxx Farmacia (India) Lip	Misomax
Medley Pharmaceuticals	Clear
Neclife/Neccare	Nectrapill
Neiss Labs Ltd	Abortom Kit
Orchid Chemicals & Pharmaceuticals Ltd	Dismis MM Kit
Pride Healthcare	Pregyne Kit
Rainbow/Naari for export	Safe-T Kit
Remedial Healthcare	She-Bort
SandMartin Pharmaceuticals Pvt Ltd	Vinmis
Smayan healthcare Pvt Ltd	Ucomif-CombiKit
Sun Pharmaceutical Industries Ltd	MifeprinKit
Syn Labs	Syn-Bort
Synokem	A-Kare/ Safe Abort Kit/ Safe n Sure Kit
Theogen Pvt Ltd	Avort
Theon Pharmaceuticals Ltd	Combi Kit
Torrent pharmaceuticals	Herwont kit
Unimarck Healthcare Ltd	MTP
Voizmed	V-Bort Kit
Zydus Cadila	MifegestKit
Mifepristone	
Abbott Healthcare Pvt Ltd	Mefipil
Alkem Laboratories Ltd	Termipil
Aristo Pharmaceuticals Pvt Ltd	Mifty
Cipla Ltd	MTPill
Coles Pharma	Colestone
Cure Quick Pharmaceuticals	Abo Pill
Bestochem Formulations Ltd	T-Pill
Bharat Serum & Vaccines Ltd	Abortab
East African (India) Overseas Ltd	Miferiv
Elder Pharmaceuticals Pvt Ltd	Elmif
Emcure Pharmaceuticals Ltd	Empri
FDC Limited	Undo
Fourrts Laboratories Pvt Ltd	Mefetrac
HLL Lifecare Limited	Mifepro
Intas	AntiPreg
Lupin Ltd	Lupin Ltd
Mankind Pharma Ltd	Unwanted
Medipol Pharmaceuticals India Pvt ltd	Mifepristone
Novaduo Pharma	Mistone
Profic Organic Ltd	Cedate
Sun Pharmaceutical Industries Ltd	Medabon* MifePrinKit
Synokem Pharmaceuticals Ltd	MFT
Taj Pharmaceuticals Ltd	Mifebort
Zee Laboratories Ltd	Relezed
Zydus Cadila	Mifegest
Misoprostol	
Abbott	Mesopil
Acme	Misoclear^¥^
BestoChem Formulations India Ltd	Miso
Cipla	Misoprost^¥^
Mankind Pharmaceuticals	Prestakind
Naari Pharma Pvt Ltd	Miso-Kare
Ridley Life Science Pvt Ltd	Misorest
Sun Pharmaceuticals	Zitotec
Vivek Pharmachem India Ltd	Misoprostol
Zydus Cadila	Cytolog

*Medabon is SRA-approved

^¥^Misoclear and Misoprost are WHO Prequalified

#### Quality assurance

State FDCAs in India oversee regular inspections of manufacturing sites for compliance with current good manufacturing practice (cGMP) and for monitoring adverse reactions. In India, there are two basic approvals for manufacturing facilities. One is cGMP determined by state FDCA inspections and based on the inspection risk-assessment. The other is cGMP determined by joint inspection by CDSCO Zonal Officers and inspectors using a quality risk approach and a checklist relying on the WHO cGMP scheme for pharmaceutical products, and on national Certificates of Pharmaceutical Product, also based on WHO guidelines. Both types of inspections happen approximately every three years. For hormonal products like, misoprostol, to align with WHO cGMP standards, manufacturers must maintain a dedicated hormonal facility for production.

In public sector procurement systems, quality assurance, three random samples from manufacturing sites or pharmacy registers are selected at the central warehouse. These are then dispatched to the Head Office of Drug Control and then subsequently sent to three different labs for analysis. Should medicines fail to meet assay or pharmacopeia standards, district warehouses receive directives to freeze the stock until the Quality Control Department issues further directions on next steps [22].

### Policy and financing

Abortion care is integral to India’s Reproductive, Maternal, Newborn, Child and Adolescent Health (RMNCAH) strategy and is incorporated into national service delivery guidelines and the National Health Mission (NMH) Program Implementation Plans [10]. Although robust national policies exist and guidelines on provider eligibility, capacity building and financial support for MA medicine procurement are already in place [1, 11, 12] state-level variations persist.

The national essential medicines list (EML) guides public sector procurement, but states have autonomy to formulate their essential medicine lists based on the specific requirements. This assessment identified that while national policy and inclusion of mifepristone and misoprostol exists, translation into state EMLs is inconsistent. For example, in Tamil Nadu, the 2019–2020 EML includes misoprostol (200 mcg tablet) and mifepristone on a separate “Specialty Drug List” for public tender – a temporary designation for newly added medicines. In Rajasthan, the 2020 EML includes mifepristone tablets for primary health centers and misoprostol tablets for sub-centers and above. However, the MTP combi-pack, not in the EML, is on an approved rate list and is procured by the Government of Rajasthan [23].

### Procurement and distribution

Public sector procurement of MA medicines is devolved to the state level; there is no centralized procurement system at the national level. State governments have established corporations to procure essential medicines through bulk purchasing utilizing their NHM funds.

These funds are allocated based on Program Implementation Plans submitted by the states. During the fiscal year 2019–2020 and 2020–2021 under the budget head of ‘Drugs for Safe Abortion’ varies and is contingent on state needs and the availability of existing stock; budget needs and allocations are generally based on previous trends. Financing (including procurement) of MA medicines varies significantly across states despite national level efforts to allocate funds to purchase MA medicines.

The variation in requirement also poses challenge for procurement. States with small quantity tender volumes may not be attractive to the large manufacturers for competing for tenders. A review of a sample of public tenders at state level reveals that there is variation in combi-pack tender volumes across states. For example, in Rajasthan and Tamil Nadu, the tender volume is on average 17,000 and 15,000 combi-packs, respectively, compared to larger public-sector tenders in states like Madhya Pradesh (80,000) and Karnataka (40,000).

Besides small tender volumes, there are also other factors which dissuade commercial manufacturers from competing for public tenders, such as onerous paperwork, payment delays, and preference for in-state suppliers to promote local businesses. The non-governmental sector has an important role in procurement and distribution of MA medicines through public–private partnerships.

MA medicines are also available for purchase upon prescription through retail pharmacies. There is wide variability in the availability of MA medicines in pharmacies across states. A large-scale medicine survey of essential medicines conducted by the MoHFW in 2014–16 found that misoprostol has limited availability in retail outlets [24]. Data from NGO pharmacy surveys indicate that chemists cite strict monitoring and reporting by state-level FDCAs as reasons for ceasing to stock and dispense MA medicines in pharmacies [23–25]. Key informant interviews in both states corroborate the finding that documentation requirements for pharmacists (copies of prescriptions on file, client signatures) and the risk of loss of license, create barriers to pharmacists’ willingness to stock MA medicine. Pharmacies affiliated with hospitals with obstetricians and gynecologists are most likely to stock MA medicines.

The national health survey clearly shows that the majority of abortions are performed in the private health sector (53%), and only 20% in the public health sector [4]. There is a robust private sector market for MA medicines in India with not only commercial distributors but also social marketing organizations distributing and selling MA medicines through a variety of private sector providers, outlets, clinics, and pharmacies [25].

### Provider knowledge and behavior

The MTP Act defines who can provide abortion in India [1, 11]. Medical doctors are permitted to perform MTP up to 20 weeks’ gestation if they have a post-graduate degree or diploma in obstetrics and gynecology, have completed six months of residency in obstetrics and gynecology or have at least one-year experience in the practice of obstetrics and gynecology. Physicians with a Bachelor of Medicine, Bachelor of Surgery (MBBS) are only permitted to provide first trimester (up to 12 weeks’ gestation) MTP/CAC services after completing training at a government approved training center or hospital and becoming a certified provider [12].

Funding for the training of these providers is allocated under the NHM Program Implementation Plan which primarily targets training master trainers and Medical Officers. However, CAC training is inconsistent, highlighting the need for optimized fund utilization. Interviews with providers highlighted the need for innovative approaches to teaching and learning without diverting providers from service delivery.

The study reveals a significant gap in providers’ awareness of the updated national abortion guideline, and the abortion law in India [11, 26]. Although the combi-pack is approved for use up to nine weeks (63 days) of gestation, many providers limit its use to seven weeks (49 days) of gestation, per the MTP Act. Additionally, invasive practices, like dilatation and curettage, are still commonly practiced [4, 26–29]. Key informant interviews showed that knowledge of the WHO Abortion Care guideline is inconsistent. There is lack of alignment between WHO recommendations for use of MA medicines, which is up to 12–13 weeks of gestation [2, 3], the combi-pack regulatory approval (9 weeks) and the MTP Act. This creates challenges in evidence-based decision-making in abortion care services, particularly in private sector, which often sees minimal regulatory supervision.

### End user knowledge and behavior

This assessment, primarily based on literature review, indicates a significant knowledge gap regarding abortion legality and availability of safe services, particularly among young and unmarried [4]. The stigma surrounding abortion is pronounced, especially in rural areas, and the emphasis on sex-selective abortion has fostered a widespread belief that all abortions are illegal [4, 28, 29]. Despite free services in the public sector, many abortions take place in private sector at considerable out-of-pocket costs (retail cost ranges from 335–600 INR, equivalent to USD$4-$7.25), due to unawareness about free services in public sector [30, 31]. Woman’s negative perceptions of the legality, quality, privacy and confidentiality, and hostile public facility environment deter women from accessing public sector services, often resulting in unsafe abortion practices [4, 30–32].

Community awareness activities about safe abortion services is not uniformly prioritized and varies across the state-level. The Reproductive, Maternal, Newborn, Child and Adolescent Health program calls for, “Routine orientation and training of Accredited Social Health Activists to equip them with skills to create awareness about abortion issues in the community and facilitation of women's access to services”, yet dedicated funding for this initiative is often absent.

## Discussion and recommendations

This paper is the first landscape assessment of MA medicines, particularly the combi-pack, using the WHO framework for assessing the availability of MA medicines. This is a standardized, evidence-based approach that not only identifies areas of intervention along the supply-chain but also provides significant opportunities for establishing India’s potential in providing quality-assured MA medicines for the Region and the world.

India is an important manufacturer of MA medicines [22, 23]. However, we found that uniform implementation of quality assurance and regulatory procedures requires strengthening at the national and state-levels. This is well within the scope of authority of the national regulatory body. While CDSCO oversees and coordinates state FDCA more nuanced attention on MA medicines is needed to reduce the variation between the enforcement and uptake of regulatory standards. This, combined with a lack of structured norms defining state EMLs and the national EML, creates discrepancies that add undue barriers to public sector procurement and availability of MA medicines.

Combi-packs in India are approved by state-level regulatory agencies with oversight by CDSCO. Manufacturing a quality misoprostol product is challenging as pure misoprostol is extremely unstable and easily degraded by moisture [33–35]. While requirements for the quality testing of MA medicines are in place, including during manufacturing and immediately post-procurement, this may not be sufficient to determine a level of quality of MA products that can be upheld up to international standards. Medicines procured by state agencies have been found to be monitored for their quality prior to distribution for public sector facilities. However, state procurement agencies may miss potential quality issues of combi-pack and misoprostol tablets with only baseline testing upon receipt of medicines at warehouses [13, 33]. To improve overall quality of MA medicines, quality control should be replaced by quality assurance at all stages of manufacturing and strengthened implementation of cGMP should be ensured across manufacturing sites.

WHO provides technical assistance to local manufacturers, including capacity building for cGMP, to support a WHO-PQ application process and foster local production. MA medicine manufacturers could apply for WHO PQ either for finished pharmaceutical products, which would facilitate procurement of their medicines outside of India, or for or active pharmaceutical ingredients, which would support manufacturing of quality medicines internationally. Manufacturers could also consider applying for approval from additional SRAs, if they have the manufacturing capacity and meet CDSCO export conditions, to cater to the global demand for MA medicines.

Procurement of MA medicines by states does not guarantee availability of MA medicines at public facilities or public sector pharmacies. Regional tendering for procurement may create a market size that would entice small and mid-level manufacturers to compete. Specific interventions at the state-level, such as streamlining the payment process, e-tendering, and capacity building for tendering processes and streamlining supply chain aspects and other initiatives may overcome the barriers dissuading commercial manufacturers from competing for tenders. Public sector procurement capacity should be strengthened to ensure that enough combi-packs are available at approved MTP public sector sites. All states should capitalize upon available resources for MA medicine and improve the quantification and forecasting of MA medicines.

Since 2015, WHO has published or updated multiple guidelines with new and rigorous evidence which could be considered for national uptake [2, 3, 33–36]. The updated MTP amendment provides an opportunity for updating and aligning national guidelines in India with global evidence-based recommendations. CAC training, in addition to being skill-based, must also be designed to clarify values, address social stigma, and provide comprehensive abortion services care, with quality, safety and most importantly respect [37–39]. Finally, states may introduce focused awareness campaigns to address the inaccurate conflation of laws and its effect on the correct provision of abortion care and invest in raising community awareness about the legality of abortion and availability of free safe abortion care service in public health facilities.

### Limitations and strengths

This assessment provides the first overview of the MA medicines landscape in India. The implementation of a previously tested WHO framework in other countries creates structure and comparability across contexts [17]. Furthermore, the methodology can be easily replicated to determine changes in the landscape. A limitation to the assessment, due to the COVID-19 pandemic, was that interviews were largely conducted virtually or by telephone. Validity to determine the extent to which it accurately measures what it intends to measure was beyond the scope of the current paper.

Some interviews, notably with Food and Drug Control Administrations, could not be secured, limiting information collected to publicly available documents and expert feedback. This is relevant as the current registration status of the products listed in Table 1 is devolved to the state-level and could not be verified. End-user knowledge and behaviors could not be assessed owing to the pandemic. This may have limited the capture of the full spectrum of availability and quality of MA medicines in India. Finally, this assessment did not include analysis of export functions, current export volume, and manufacturers that export medicines, it was focused on domestic policy, supply, and procurement only.

## Conclusion

India benefits from strong national policies that center abortion care within the National Health Mission, its budgets and guidelines. India's prominence as a leading manufacturer of MA medicines guarantees a steady product supply. However, there is variation across states and this landscape assessment uncovers opportunities to enhance the availability of quality MA medicine in India. These opportunities include uniform implementation of regulatory standards, prioritizing quality-assurance during manufacturing process especially for misoprostol and establishing standardized procurement and supply chain norms across all states. Streamlining implementation efforts on laws, policies and guidelines governing MTP and MA, with regular in-service training of providers on medical abortion provision in line with the latest national guidelines is required. Additionally, evidence dissemination and regular community engagement about the recently amended abortion law is needed.

## Data Availability

The data that support the findings of this study are available from the corresponding author, LL, upon reasonable request.

## Abbreviations

API: Active pharmaceutical ingredient
CDSCO: Central Drugs Standard Control Organization
CAC: Comprehensive Abortion Care
cGMP: Current good manufacturing practice
EML: Essential Medicines List
FDCA: Food and Drug Control Administrations
MA: Medical Abortion
MBBS: Bachelor of Medicine, Bachelor of Surgery
MTP: Medical Termination of Pregnancy
MoHFW: Ministries of Health and Family Welfare
NHM: National Health Mission
NRA: National Regulatory Agency
RMP: Registered medical practitioner
RMNCAH: Reproductive, Maternal, Newborn, Child and Adolescent Health
SEARN: Southeast Asia Regulatory Network
SRA: Stringent Regulatory Authority
WHO: World Health Organization
WHO PQ: WHO Prequalification

## Declarations

### Disclaimer

The views expressed in this article are those of the authors and do not necessarily represent the views of, and should not be attributed to, the World Health Organization. The mention of specific companies or of certain manufacturers’ products does not imply that they are endorsed or recommended by WHO in preference to others of a similar nature that are not mentioned. Errors and omissions excepted; the names of proprietary products are distinguished by initial capital letters.
